# Professional practice models for nurses in low-income countries: an integrative review

**DOI:** 10.1186/s12912-015-0095-5

**Published:** 2015-08-21

**Authors:** Njoki Ng’ang’a, Mary Woods Byrne

**Affiliations:** Center for Children & Families, School of Nursing, Columbia University, New York, NY USA; International Organization for Women and Development, Rockville Centre, NY USA

## Abstract

**Background:**

Attention is turning to nurses, who form the greatest proportion of health personnel worldwide, to play a greater role in delivering health services amidst a severe human resources for health crisis and overwhelming disease burden in low-income countries. Nurse leaders in low-income countries must consider essential context for nurses to fulfill their professional obligation to deliver safe and reliable health services. Professional practice models (PPMs) have been proposed as a framework for strategically positioning nurses to impact health outcomes. PPMs comprise 5 elements: professional values, patient care delivery systems, professional relationships, management approach and remuneration. In this paper, we synthesize the existing literature on PPMs for nurses in low-income countries.

**Methods:**

An integrative review of CINAHL-EBSCO, PubMed and Scopus databases for English language journal articles published after 1990. Search terms included *nurses, professionalism, professional practice models, low-income countries, developing countries* and relevant Medical Subject Heading Terms (MeSH).

**Results:**

Sixty nine articles published between 1993 and 2014 were included in the review. Twenty seven articles examined patient care delivery models, 17 professional relationships, 12 professional values, 11 remuneration and 1 management approach. One article looked at comprehensive PPMs.

**Conclusions:**

Adopting comprehensive PPMs or their components can be a strategy to exploit the capacity of nurses and provide a framework for determining the full expression of the nursing role.

## Background

The global health workforce deficit projected to reach 12.9 million personnel by 2035 presents both an opportunity and a dilemma for nurses worldwide, but especially for colleagues in low-income countries [1]. With more than 35 million nurses comprising the greatest proportion of health personnel globally, members of the profession are strategically positioned to contribute significantly to health services delivery [2]. Organizational systems - structures, processes and values-create a blueprint to guide professional nursing practice; without proper organizational systems, nurses cannot optimize patient surveillance and deliver interventions safely and reliably [3, 4]. Nurses in low-income countries contend with an overwhelming disease burden and persistent health human resources crisis that manifests in deep personnel shortages, inappropriate skill mix and maldistribution of health workers [5, 6]. Yet, the state of organizational systems in low-income countries, which form the essential context for professional nursing practice, has not been fully examined in spite of a robust discourse on strengthening the capacity of nurses in these regions. With an urgent global agenda exerting pressure to curb preventable and premature mortality, nurses in low-income countries facing worsening health workforce shortages over the next 20 years are compelled to find ways to mobilize and meet the demands of a rapidly evolving health services delivery milieu.

Professional practice models (PPMs) have been proposed as a means of instilling organizational systems that mobilize nurses by granting them control over delivery of patient care and the overall work environment [7]. Hoffart and Woods posited that PPMs encompass five essential building blocks: professional values, patient care delivery systems, professional relationships, management approach and remuneration [7]. Professional values are the central tenets that guide professional nursing practice and form a foundation for the other elements of a PPM [7]. The nursing code of ethics constitutes one type of professional value [7]. Patient care delivery systems signify the manner in which responsibility for the gamut of patient care duties is configured [7]. One example of a patient care delivery system is the delineation of nursing roles from non-nursing roles [7]. Professional relationships refer to nurse-to-nurse interactions and exchanges between nurses and other members of the multidisciplinary team that are essential for effective collaboration on patient-related matters [7]. The management approach is concerned with the decision making structures and processes employed in an organization [7]. Finally, remuneration describes how nurses are compensated and rewarded in recognition of their performance [7].

Attaining simultaneously all five components of a PPM is difficult regardless of high- or low-income country status. Worldwide, only 400 select hospitals located in Australia, Lebanon, Singapore, the United Kingdom and the United States have succeeded in implementing the most prominent example of a PPM, the Magnet® model [8]. In Magnet®-designated facilities, the signature characteristic is nurses’ representation in all hospital affairs; this includes a visible nursing leadership, autonomous nursing care, collaborative nurse-physician relationships and opportunities for professional development [9]. Facilities that have achieved the highly coveted Magnet® designation exhibit higher levels of patient and nurse satisfaction as well as significantly lower rates of morbidity and mortality [10, 11].

The case of more than 30,000 nurses in 12 European countries responding to the RN4CAST survey demonstrates how professional nurses and nursing practice are undermined when the ideals of a PPM are unevenly implemented or unavailable [4, 12]. More than a third of nurses reported that opportunities for career advancement were absent in their facilities (range: 33 % in Switzerland to 84 % in Spain) [12]. More than half of nurses in 11 of the 12 countries reported lack of opportunities to participate in policy decision making (range: 63 % in the Netherlands to 88 % in Spain) [12]. More than half of all nurses in the 12 countries disagreed with the item *enough nurses on staff to provide quality patient care* (range: 52 % in Switzerland to 85 % in Poland). In addition, the researchers showed that in 6 of the 12 countries, more than half of nurses perceived their chief nursing officers not to have equal standing with other high level hospital executives (range: 51 % in Finland to 82 % in Sweden) [12].

Practice environments or facilities that deny nurses PPMs – which confer authority over the environment of care, including to make appropriate and timely care related decisions in response to changes in patient conditions – are problematic because quality of care can be compromised leading to adverse outcomes [13]. European nurses in the RN4CAST study acknowledged leaving important patient care related tasks undone due to a burdensome workload and time constraints [12]. At least one third of nurses in Germany, Greece, the Netherlands and Spain rated the quality of care in their wards as poor or fair [4]. Up to two thirds of nurses in the RN4CAST study were not confident that patients could manage their own conditions upon discharge [4].

Similar lapses in care have been reported in low-income countries. In India, for example, nurses working in New Delhi maternity homes attributed impolite and disrespectful treatment of impoverished women to long hours, poor pay and overcrowding of facilities [14]. In turn, the women shunned safer facility deliveries in favor of childbirth at home supervised by traditional birth attendants with little or no training to identify complications and implement necessary interventions [14]. PPMs provide nurses with the necessary infrastructure to fulfill their professional obligation to deliver optimal health services. Tangible improvements realized in patient outcomes, as well as in patient and nurse satisfaction, when PPMs are in place outweigh the inherent difficulties of installing them and suggest their utility even in low-income country settings. To date, this remains unexplored.

Seventy per cent of the 83 countries failing to meet the recommended level of 23 nurses, midwives and physicians necessary to provide 80 % coverage of essential services, such as attendance of childbirth by skilled personnel, are located in sub-Saharan Africa and south East Asia [1]. At the same time, 85 % of all maternal deaths aggregate in the two regions with the majority of deaths (56 %) occurring in sub-Saharan Africa [15]. Together, sub-Saharan Africa and south East Asia account for the highest incidence of new cases of HIV infection [16]. The rise of risk factors, such as hypertension, tobacco smoking and high body mass index, likely to lead to non-communicable and chronic illnesses, including cardiovascular disease and diabetes, threaten to exacerbate the existing disease burden in low-income countries [17].

An increasingly common response to meet demand for essential health services in low-income countries, such as emergency obstetric care and antiretroviral therapy (ART), requires nurses to assume an expanded role in the practice known as task-shifting. Task-shifting is defined as the transfer of responsibilities normally assigned to health personnel with advanced training to cadres with less pre-service education [18]. Focusing on nurses as essential partners in meeting global health goals is the right step – one that has been endorsed by global nurse leaders, including the newly formed *Global Advisory Panel on the Future of Nursing* (GAPFON) [19]. Yet, the extent to which organizational systems low-income countries are equipped to support nurses in fulfilling their professional obligation within under-resourced and over-stretched settings has not been fully articulated. In this paper, we propose PPMs as a framework for galvanizing the capacity of nurses and appraise the existing literature to gauge the degree to which elements of PPMs have been implemented for nurses in low-income countries.

## Methods

CINAHL-EBSCO, PubMed and Scopus databases were searched for journal articles published in English after January 1, 1990 using the following key words: nurses, professionalism, professional practice models, developing countries, low-income countries and relevant Medical Subject Heading Terms (MeSH). Low-income or developing country status was assigned based on World Bank classifications [20]. Articles were included in the review if the purpose of the paper was to describe theoretically or evaluate empirically in a low-income nation one or more elements of a PPM as defined by Hoffart and Woods [7]. Articles discussing these elements in high-income or developed countries were excluded. Also excluded were articles reporting programmatic initiatives in low-income countries where nurses have been involved but their professional development was not intrinsic to the intervention. The first author retrieved articles from the 3 databases using the predetermined search terms and selected relevant titles based on the eligibility criteria. Both authors independently assessed 20 % of the abstracts for eligibility allowing for measures of agreement and reliability between the two researchers to be calculated. The resulting inter-rater agreement of 86 % and Cohen’s kappa of 0.73 were judged sufficiently high to allow only the first author to proceed with the selection procedure. Any disagreements were resolved by consensus. The quality of each article was quantified by a score of 0 or 1 (low or high) assigned by consensus on four criteria: authenticity, informational value, methodological quality and representativeness [21, 22]. Data analysis comprised categorizing articles according to year of publication, study methodology used and the country in which the research was conducted. Then studies were clustered according to the element of a PPM discussed and results synthesized to elucidate the state of the evidence on PPMs for nurses in low-income countries. We applied to this integrative review the same standards of rigor reserved for primary research [23].

## Results

The initial search led to more than 20,000 articles. Query limits applied to enhance the specificity of this initial search included the terms *professional values, code of ethics, patient care, care delivery systems, management approach, decision making, professional relationships, interdisciplinary relationships, salary* and *compensation* [24]. The high specificity of the augmented search did not correspond to a high sensitivity, which meant numerous articles captured using the initial search terms were excluded [24]. The tradeoff was to proceed with the initial time-consuming search strategy that ensured all relevant articles meeting specified criteria were included.

**Fig. 1:**
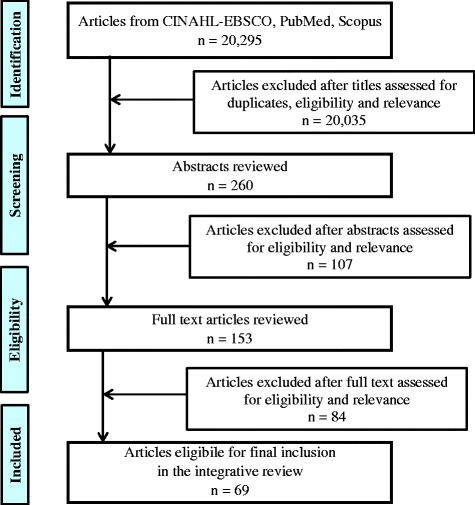
Schematic representation of article selection process

Titles of the 20,295 articles retrieved using the initial search terms were scanned for key words relevant to the eligibility criteria outlined previously. After 20,035 duplicates and non-eligible titles were put aside, the abstracts of the remaining 260 titles were extracted. From these, 153 full text articles were retrieved and assessed for eligibility and relevance. Eighty four articles did not meet the eligibility criteria when the full text was reviewed and were subsequently eliminated from the review. The remaining 69 articles met the eligibility criteria and were included in the integrative review. The article selection process is represented schematically in Fig. 1.

**Fig. 2:**
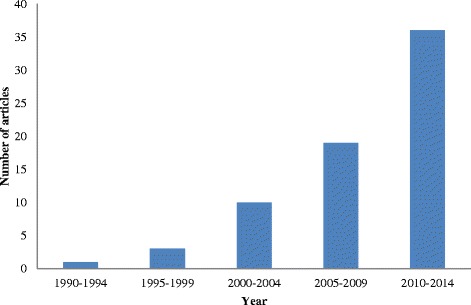
Distribution of selected articles by year of publication

**Fig. 3:**
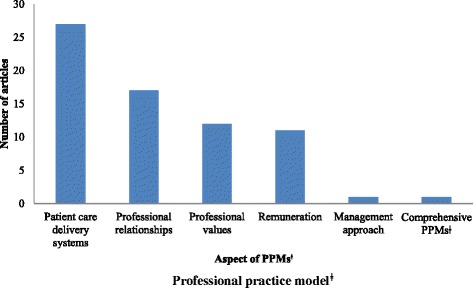
Distribution of articles according to element of PPMs^ǂ^ addressed. ^ǂ^ Professional practice model

The majority of articles (*n* = 19) examined aspects of PPMs in the World Health Organization (WHO) sub-Saharan Africa region compared to 14 in the South-East Asia region, 9 in the Eastern Mediterranean region, 8 in the Western Pacific region, 6 in the Americas and 1 in the European region. Twelve articles addressed PPMs in more than one region or country. The studies we analyzed applied a myriad of quantitative and qualitative research methodologies. A summary of the results is presented in Table 1. The first study was published in 1993 and almost all (*n* = 65) were published in the 2000s, with the period after 2010 accounting for 52 % of articles as is depicted in Fig. 2. Only 1 article dealt with PPMs as a comprehensive entity; the other 68 addressed one or more, but not all, of the individual elements encompassed in the model put forward by Hoffart and Woods [7]. The distribution of articles according to the element of a PPM addressed is presented in Fig. 3. The study outcome was literature describing or examining PPMs for nurses in developing countries. We have grouped our findings according to the elements of a PPM described by Hoffart and Woods [7].

**Table 1:** Summary of selected articles

Author	Year	Country	Aspect of PPMs^f^	Study design and sample	Study aims and context
Akinsola et al. [29]	2001	Botswana	Professional values	Literature review	Explore ethical dilemmas faced by nurses
				Peer-reviewed journal articles, international and national grey literature	Rural settings
Botes [28]	1999	South Africa	Professional values	Case study	Explore ethical dilemmas faced by nurses
					Low-resource settings
Donkor et al. [25]	2011	Ghana	Professional values	Cross-sectional survey	Describe nurses’ approach to ethical dilemmas
				Nurses attending an international conference (*n* = 200)	
				Purposive sampling	Various practice settings in Ghana
Donkor et al.^d^ [30]	2011	Ghana	Professional values	Literature review	Describe the challenges of nursing practice in Ghana
				Peer-reviewed journal articles, international and national grey literature	
Harrowing et al. [26]	2011	Uganda	Professional values	Critical ethnography	Describe the impact of moral distress amongst providers of HIV/AIDS care
				Focus groups, interviews and participant observation	
				Acute care and public health nurses (*n* = 24)	1 tertiary care facility
Jewkes et al. [31]	1998	South Africa	Professional values	Ethnography	Explore health seeking practices of pregnant women
				Focus groups, interviews and participant observation	
				Midwives, nurses, family planning advisor (*n* = 13)	Public maternal health facilities
				Patients utilizing maternal health services (*n* = 90)	
Kaeswarn et al. [27]	2003	Thailand	Professional values	Cross-sectional survey	Examine impact of nurses’ beliefs about postpartum care on nursing practice
				Nurses (*n* = 372)	
Mill et al. [34]	2013	Jamaica	Professional values	Participatory action research	Explore the role of stigma on nursing care delivered to People Living with HIV/AIDS
		Kenya		Interviews and focus groups	
		South Africa		Purposive sampling	
		Uganda		Nurses and midwives (*n* = 84)	
Pelzang et al. [35]	2010	Bhutan	Professional values	Mixed methods	Explore understanding and implementation of patient-centered care
				Cross-sectional survey and open-ended questionnaire	
				Purposive sample	
				Nurses (*n* = 87)	Secondary and tertiary facilities
Shields et al.^e^ [32]	2003	Indonesia	Professional values	Literature review	Describe the context of nursing practice in Indonesia
				Peer-reviewed journal articles, international and national grey literature	
Shields [33]	2005	Multiple	Professional values	Editorial	Discuss the ethical dilemmas faced by nurses practicing in under-resourced settings
Tschudin et al. [36]	2003	Multiple	Professional values	Literature review	Examine the ethical implications of war and conflict on nursing practice
				Peer-reviewed journal articles, international and national grey literature	
Alberto et al. [37]	2014	Argentina	Patient care delivery systems	Medical record review	Describe the role of the ICU liaison nurse
				Content analysis of nursing exemplars	
				Intensive care unit (ICU) liaison nurses (*n* = 5)	Intensive care unit in 1 tertiary care facility
Alquidimat et al. [39]	2009	Jordan	Patient care delivery systems	Case study	Describe the role of the clinical nurse coordinator
					Pediatric cancer center
Araya et al.^a,c,d^ [53]	2009	Ethiopia	Patient care delivery systems	Cross-sectional survey	Describe the experience of mental health nurses and perceptions of mental health service provided by nurses amongst interdisciplinary colleagues
				Nurses (*n* = 42) and their interdisciplinary colleagues (*n* = 55)	
					18 hospitals and 4 health centers
Barrett et al. [54]	2009	Multiple	Patient care delivery systems	Open-ended questionnaire	Identify key issues encountered by mental health nurses
				Qualitative thematic analysis	
				Subscribers to an online forum coinciding with the release of the Nurses in Mental Health Atlas (*n* = 615)	
					80 countries
Choromanski et al. [62]	2012	Multiple	Patient care delivery systems	Tool development	Develop an International Classification of Nursing Practice® subset for documenting nursing care provided to children with HIV/AIDS
					Low-income country settings
Colquhoun et al. [46]	2012	Solomon Islands	Patient care delivery systems	Qualitative thematic analysis	Describe the role and context of pediatric nursing
				Semi-structured interviews	Primary, secondary and tertiary facilities
				Pediatric nurses (*n* = 21)	
Day et al.^a,c^ [47]	2008	Guatemala	Patient care delivery systems	Review of medical records, policies, procedures and job descriptions	Evaluate quality of nursing practice based on Joint Commission International standards
					St. Jude’s Children’s Research Hospital international outreach site
				Interviews and participant observation	
Day et al.^a,c^ [64]	2013	Guatemala	Patient care delivery systems	Review of medical records, policies, procedures and job descriptions	Evaluate quality of nursing practice based on Joint Commission International standards
					St. Jude’s Children’s Research Hospital international outreach site
				Interviews and participant observation	
De Silva et al. [50]	2010	Sri Lanka	Patient care delivery systems	Ethnography	Explore nursing management of cancer pain
				Qualitative thematic analysis	
				Interviews, participant observation and journal/field notes	
					1 medical/cancer ward
				Nurses (*n* = 10)	
Ersser et al. [41]	2000	Multiple	Patient care delivery systems	Literature review	Outline the vision of the International Skin Care Nursing Working Group to promote global skin health
				Peer-reviewed journal articles, international and national grey literature	
Hoyt [65]	2006	Multiple	Patient care delivery systems	Program description	Describe the experience of implementing Problem Solving for Better Health Nursing methodology
					15 countries
Jejeebhoy et al. [43]	2011	India	Patient care delivery systems	2-sided equivalence study	Compare level of safety and efficacy between manual vacuum aspiration (MVA) performed by nurses and MVA performed by physicians
				Nurses (*n* = 10) and physicians (*n* = 10)	5 non-governmental reproductive health clinics
Kep [52]	2012	Papua New Guinea	Patient care delivery systems	Editorial	Perspective on changes in nursing practice over 30 years
Lu [61]	2007	China	Patient care delivery systems	Cross-sectional survey	Explore perceptions of the nursing role
				Nurses (*n* = 512)	Medical/surgical departments in 2 teaching hospitals
Miles et al. [55]	2006	Multiple	Patient care delivery systems	Literature review	Describe issues surrounding medication prescribing by nurses
				Peer-reviewed journal articles, international and national grey literature	
					Low-income countries
Miles et al. [56]	2007	Botswana	Patient care delivery systems	Case study	Highlight the case of anti-retroviral roll-out in Botswana to make the case for shifting to nurse-led models in HIV/AIDS care
				Literature review	
				Peer-reviewed journal articles, international and national grey literature	
Mweemba [44]	2003	Zambia	Patient care delivery systems	Editorial	Perspective on the challenges encountered by acute care and public health cardiovascular nurses
Nankumbi et al.^a,c,d^ [57]	2011	Uganda	Patient care delivery systems	Mixed methods	Evaluate a new model of HIV/AIDS care
				Cross-sectional survey	
				Key informant interviews	
				Qualitative thematic analysis	6 urban government clinics
				Nurses (*n* = 20) and nurse managers (*n* = 6)	
Paul et al. [42]	2013	India	Patient care delivery systems	Mixed methods	Compare efficacy of and patient satisfaction with nurse-led epilepsy follow-up care to that of a physician
				Cross-sectional survey	
				Semi-structured interviews	
				Nurse (*n* = 1) and physician (*n* = 1)	Outpatient neurology clinic
Plager et al. [59]	2009	Madagascar	Patient care delivery systems	Needs assessment	Describe strategies to improve nursing education and advance the profession
				Interviews and site visits	
Premji et al. [40]	2013	Multiple	Patient care delivery systems	Literature review	Examine the state of neonatal nursing in low-income countries
				Peer-reviewed journal articles, international and national grey literature	
Rukanuddin [63]	2005	Pakistan	Patient care delivery systems	Tool development	Describe the process of developing and testing International Classification of Nursing Practice® subsets for documenting maternity and cardiology nursing care
Scott et al. [45]	2012	South Africa	Patient care delivery systems	Key informant interviews and focus groups	Explore perspectives of nurses and middle managers on new staffing procedures
				Qualitative thematic analysis	6 primary care clinics
				Nurses (*n* = 42) and managers (*n* = 12)	
				Purposive selection of facilities	
Sharma et al. [58]	2013	India	Patient care delivery systems	Grounded theory	Describe the scope of nursing practice in obstetric settings
				Semi-structured interviews and participant observation	
				Nurses (*n* = 10), Physicians (*n* = 9), Midwives (*n* = 4), Nursing and midwifery faculty (*n* = 4), Student (*n* = 1)	
					1 tertiary, 1 secondary and 3 primary facilities
				Purposive selection of facilities	
Squires et al.^c^ [48]	2012	Mexico	Patient care delivery systems	Qualitative content analysis	Examine nurses’ perspective of their work environment
				Semi-structured interviews	
					Diverse practice settings (acute care, community, academia)
				Nurses (*n* = 46)	
				Geographic locations selected purposively	
Uebel et al. [51]	2013	South Africa	Patient care delivery systems	Meta-ethnography of 3 studies	Explore factors driving integration of HIV/AIDS services into primary care clinics
				In-depth interviews, focus groups and participant observation	
				Nurses (*n* = 44), other stakeholders (*n* = 32) and patients (*n* = 27)	More than 40 clinics
Walani [38]	2006	Pakistan	Patient care delivery systems	Case study	Describe the nurse case manager role in medical/surgical units
					1 university medical center
Bender et al. [74]	2011	Ethiopia	Professional relationships	Case study	Describe Ethiopian-Canadian collaborative research project examining intimate partner violence
Brown et al. [73]	2013	Indonesia	Professional relationships	Case study	Describe Indonesian-Australian collaborative program to enhance clinical skills
Coverston et al. [70]	2004	Guatemala	Professional relationships	Qualitative content analysis	Explore factors that attract and retain nurses to the profession
				Nurses (*n* = 5)	
El-Jardali et al.^d^ [68]	2011	Lebanon	Professional relationships	Mixed methods	Explore the relationship between nursing work environment and intention to leave
				Cross-sectional survey	
				Open ended questionnaire	
				Qualitative thematic analysis	69 hospitals with at least 20 beds
				Nurses (*n* = 1793)	
George et al. [81]	2013	Multiple	Professional relationships	Systematic literature review	Evaluate the degree of collaboration between counterparts in low- and high-income countries
				Peer-reviewed journal articles (*n* = 9)	
Hendel et al. [80]	1996	Multiple	Professional relationships	Case study	Describe networking and training program in Israel for perioperative nurses from low-income countries
Jones et al. [60]	2000	Vietnam	Professional relationships	Case study	Describe nursing practice and education
Lasater et al. [76]	2012	Cambodia	Professional relationships	Case study	Describe United States-Cambodia collaborative program to boost professional nursing
McInerney [71]	1993	Uganda	Professional relationships	Editorial	Perspective from a short-term international volunteer experience
Mosby et al. [77]	2008	Central America	Professional relationships	Case study	Describe Central American-United States collaboration to address nutritional management of children with cancer
Papastavrou et al.^e^ [69]	2012	Turkey	Professional relationships	Cross-sectional survey	Examine perceptions of the professional practice environment
				Convenience sampling	
				Nurses (*n* = 156)	
					Orthopedic surgical wards in 7 tertiary or private facilities
Schaepe et al. [82]	2011	Uganda	Professional relationships	Ethnography	Describe the role of palliative care nurses
				Semi-structured interviews, participant observation and field notes	
				Nurses (*n* = 20)	
Silinzieds et al. [79]	2012	Nepal	Professional relationships	Case study	Describe Nepalese-Australian collaborative program to improve the quality of nursing care of patients undergoing plastic and reconstructive surgery
Sudhaker [83]	2008	India	Professional relationships	Letter to the editor	Describe participatory research project aimed at empowering nurses to curb hospital acquired infections in acute care facilities
Thomson et al. [78]	2008	Sri Lanka	Professional relationships	Case study	Describe Sri Lankan-United Kingdom collaborative program to improve quality of diabetes care
Walusimbi et al. [72]	2002	Uganda	Professional relationships	Editorial	Describe Ugandan-United States collaborative program to improve HIV/AIDS care
Wraa [75]	2013	Nepal	Professional relationships	Case study	Describe Nepalese-United States collaborative program to improve quality of post-anesthesia nursing care
Anonymous [88]	2010	India	Remuneration	Editorial	Speech delivered by the vice-President of India during national nursing award ceremony
Anonymous [89]	2010	India	Remuneration	Editorial	Speech delivered by the Indian Minister of Health & Family Welfare during national nursing award ceremony
Delobelle et al.^a^ [66]	2010	South Africa	Remuneration	Cross-sectional survey	Examine the relationship between demographic characteristics, job satisfaction and intent to leave
				Nurses (*n* = 137)	
				Convenience sample	
					20 fixed and 6 mobile primary health clinics
Du Toit et al. [91]	2011	Western Pacific Islands	Remuneration	Qualitative situation assessment	Describe organizational elements contributing to quality of ophthalmic care delivered by nurses
				Semi-structured interviews	
				30 nursing graduates of an ophthalmology program	
El-Jardali et al. [85]	2013	Lebanon	Remuneration	Cross-sectional survey	Explore factors influencing nursing retention
				Non probability sampling	
					63 facilities serving underserved areas
				Nurses (*n* = 857)	
Hamid et al. [49]	2014	Pakistan	Remuneration	Qualitative narrative analysis	Compare perceptions of job satisfaction between nurses in public and private facilities
				In-depth interviews	
					2 tertiary, teaching facilities
				Purposive sampling	
				Nurses (*n* = 41)	
Hollup [92]	2012	Mauritius	Remuneration	Qualitative thematic analysis	Explore factors attracting and retaining nurses to the profession
				Semi-structured interviews and participant observation	
				Convenience sampling	
				Nurses (*n* = 47)	
Lu et al.^a,c^ [67]	2007	China	Remuneration	Cross-sectional survey	Examine elements of the nursing work environment
				Nurses (*n* = 512)	Medical/surgical departments in 2 teaching hospitals
Nasrabadi et al. [90]	2004	Iran	Remuneration	Literature review	Describe education, practice and research issues encountered by nurses
				Peer-reviewed journal articles, international and national grey literature	
Rockers et al. [87]	2013	Laos	Remuneration	Discrete choice experiment	Identify preferences for job characteristics amongst practicing nurses and nursing students
				Nurses (*n* = 249) and nursing students (*n* = 256)	
					Primary, secondary and tertiary facilities in 3 rural provinces
Zarea et al. [86]	2009	Iran	Remuneration	Literature review	Examine factors contributing to the nursing shortage
				Peer-reviewed journal articles, international and national grey literature	
Gulzaret et al. [84]	2011	Pakistan	Management approach	Qualitative content analysis	Explore perceptions of the community health nurse assistance manager role
				Semi-structured interviews	
				Community health stakeholders (*n* = 13)	
				Purposive sampling	
Ng’ang’a et al. [93]	2011	Multiple	Comprehensive PPMs^f^	Editorial	Promote professional practice models for nurses in low-income countries

^a^Also addresses management approach

^b^Also addresses professional values

^c^Also addresses professional relationships

^d^Also addresses remuneration

^e^Also addresses patient care delivery systems

^f^Professional practice models

### Professional values

Twelve articles looked at issues surrounding professional values, which are defined as the underlying beliefs guiding nursing practice. The literature highlighted dissonance between knowledge of ethical principles and their application in clinical practice, which was largely attributed to cultural norms and beliefs beyond the boundaries prescribed by codes of ethics, such as the International Council of Nurses (ICN) code [25–30]. These practices ranged from negating to perform appropriate evidence-based health education [27] to verbal and physical abuse of patients [30, 31]. In other reports, nurses were forced to forfeit proper procedures when inadequately staffed to manage an overwhelming patient load or sufficient quantities of medications and equipment were unavailable [26, 29, 32, 33]; these instances were said to induce moral distress on nurses [26]. Still, nurses verbalized awareness that their role was to deliver patient-centered care [26, 34, 35], but broader health systems failures were sometimes unfairly attributed to them [26]. Some authors highlighted the tension between nursing and under-resourced health systems by juxtaposing the fact that nurses in low-income countries, especially in rural areas, are subject to the same poor environments as their patients with the notion that nurses are expected to somehow overcome difficult circumstances and facilitate health [29, 31]. Calls were made for strategies to support nurses in upholding professional codes of conduct, such as value-based education [28, 31, 36].

### Patient care delivery systems

Twenty seven articles addressed patient care delivery systems or the methods applied to assign responsibility for patient care. Of these, 3 papers described development and evolution of clinical specialist and case management roles in which nurses facilitate interdisciplinary coordination of care and perform advanced assessments of adult and pediatric patients in intensive care, oncology and medical/surgical units [37–39]. International nursing associations endorsed differentiation of nursing in low-income countries into sub-specialties, such as dermatology and neonatology, as a means of enhancing quality of care [40, 41]. Evidence pointed to parity between measures of patient outcomes and patient satisfaction obtained when specialized neurologic and obstetric care was delivered by trained nurses and physicians [42, 43].

Problems with excessive workload were said to persist due to overall personnel shortages [44–46] and lack of standardized plans to match staffing to patient volume and acuity [47–50]. One paper reported a surge in workload after top-down directives to integrate HIV/AIDS care into regular clinics were implemented to scale up access to ART [51]. In facilities where workload surpassed staffing, family members assumed responsibility for activities of daily living and other nursing duties for hospitalized relatives [44, 52]. Sanctioned or sometimes unauthorized clinical practice beyond the permitted scope of nursing practice, such as prescribing medications and performing deliveries, were another response to health personnel shortages amidst overwhelming demand for services [53–58]. Potential benefits of expanded clinical roles were overshadowed by reservations about nurses’ competence and the medico-legal implications of poorly supervised and unregulated nursing practice [55, 56].

Discordance between actual and expected nursing practice was also described in terms of non-nursing roles performed by nurses [49, 59–61]. Ongoing interventions to demarcate nursing practice included reassigning “head nurse” roles performed by physicians to nurses [59] and development of international classifications of nursing practice (ICNP®) [62, 63]. Other strategies used to bolster nursing practice were alignment of patient care delivery closer to internationally accredited Joint Commission standards [64] and instilling problem solving skills that encourage nurses to take ownership of local problems by designing and implementing contextually appropriate solutions [65].

### Professional relationships

Seventeen articles examined collaboration and communication between nurses and other members of the profession as well as interdisciplinary team members. In 2 studies, nurses reported high levels of satisfaction with their nursing colleagues [66, 67]. Cases where poor relationships between nurses existed were attributed to generational differences, gender biases, divergent views held by nurses entering into practice through assorted levels of pre-service training and perceptions of favoritism by superiors [48]. Similarly, nurse-physician relationships elicited mixed reviews. While some nurses rated their relationships with physicians highly [68, 69], others described harsh treatment enabled by a wide hierarchical distance that induced subservience and intimidation to the point physicians’ actions with the potential to harm patients were overlooked [48, 60, 70].

Across borders, exemplars of relationships between nurses in high- and low-income countries established through short-term humanitarian ventures aimed at strengthening clinical and research capacity in low-income countries were abundant [47, 64, 71–80]. Challenges inherent to these international collaborations were cultural and bureaucratic differences [74] as well as lack of validated paradigms against which brief volunteerism could be modeled and measured [76]. Indeed an assessment of partnerships between nurses in high- and low-income countries found that most failed to create sustainable projects capable of thriving past the departure of high-income country partners and many did not ensure development of low-income country counterparts to their highest potential [81]. We found one example of a long-term mentorship program that has led to measurable improvements in nursing-specific quality indicators [47, 64].

Within their borders, nurses in low-income countries capitalized on their relationships with each other and with providers from other disciplines to coordinate care [47, 53, 57, 82, 83]. In one article, palliative care nurses described themselves as “spiders in a web” weaving a network between patients, other health care providers, family members, religious leaders and community volunteers [82]. Another paper reported psychiatric nurses routinely sought consults from physicians and were relied upon by other nurses to provide consultations on their patients [53].

### Management approach

Only 1 article assessed the capacity of nurses to fulfill the management role. In this study, researchers found that community health nurses were ill prepared to assume management responsibilities necessary to mobilize other health providers and translate the principles of evidence-based practice and research into meaningful changes in health services delivery [84]. Supervision activities in some settings were carried out inconsistently, which meant some nurses rarely received support from their superiors [53] and left others dissatisfied with management [66, 67].

### Remuneration

Eleven articles appraised the rewards and compensation nurses receive for their performance. While no one type or amount of compensation appealed to all nurses, low salaries were a source of dissatisfaction universally [25, 49, 53, 57, 66–68, 85, 86]. Still, some nurses expressed willingness to accept an even lower salary in exchange for non-monetary incentives, such as job security in the form of permanent employment [87]. Non-financial benefits emerged as an important source of satisfaction, including access to health care for family members, accompanying religious pilgrims as a member of the health corps, free uniforms and transportation, recognition for employees of the month [49], national Florence Nightingale Awards in commemoration of international nurses’ day [88, 89], comfortable working space, tea with sugar and adequate toilet facilities [57]. Others desired eligibility for paid vacation days, maternity leave, subsidized child care, retirement plans, low-interest loans and life-insurance policies [68, 90]. Lack of competitive salary schemes was said to negatively impact personnel retention [53] and reduce motivation to seek additional academic qualifications because salaries did not increase in tandem with added credentials [91, 92]. Job security and stable incomes associated with employment in government-run facilities were considered more desirable than private or non-governmental organization facilities whose bonus payments were sometimes dependent on periodic funding cycles and therefore not guaranteed [83, 92].

## Discussion

Our integrative review of the literature on PPMs for nurses in low-income countries provides encouraging evidence of focus and interest in examining elements within organizational systems that influence nurses and nursing practice in low-income countries. Although we discovered only one article that addressed PPMs as a comprehensive and integrated model in the low-income country context [93], it is apparent that individual components of the model have been applied, described and evaluated. Due to the heterogeneity of studies and regions assessed – as is typical of integrative reviews – the level of evidence from our review of the literature alone is not sufficient to support PPMs as a framework for configuring the nursing workforce across all low-income countries. Nevertheless, we shed light on some patterns that are worrisome and indicate the need for better organizational systems to support nursing workforce performance. Conceptually, the PPM paradigm could be such a system.

Innovations in health services delivery, such as task shifting, whose successes are largely attributed to nurses playing a leading role, relieve urgent health personnel shortages to provide quality care that is efficient, cost-effective and accessible [94, 95]. The task shifting approach executed within well designed and managed program-specific domains whereby nurses are properly supported and compensated must not be confused with circumstances in low-income countries that compel nurses to take on additional responsibilities without adequate organizational backing and with unrealistic expectations this will increase production of health services. The latter can be a double edged sword. On one hand, nurses in these settings are at the forefront of health services delivery, holding a position that would otherwise wield considerable influence over health outcomes. On the other hand, being under-qualified, ill-equipped, poorly supervised, earning meager wages and resigned to a subordinate status to physicians prevents the full expression of nursing expertise in resource deprived milieu where it is needed the most.

PPMs emphasize that clinical decisions made at the point of interface between nurse and patient mark the critical juncture at which the trajectory of illness can lead to improvements in health or worse, the cascade to death [13]. Therefore, PPMs are concerned with bolstering nurses’ surveillance of patients so that appropriate decisions are made and suitable actions taken time after time [13]. Seminal research conducted in United States facilities has shown that the odds of both failure to rescue and preventable mortality increase as additional patients are assigned to a nurse and in poor environments of care [96, 97]. One example of an inappropriate configuration of care applied in low-income countries as a result of extreme shortages of health workers that can compromise patient outcomes is the assignment of an inadequately trained nurse to be the only primary care provider serving an underserved population [98]. A qualitative investigation by Bossyns and van Lerberghe [99] found that front line nurses in Niger withheld referring patients to higher levels of care, even when those patients faced life threatening emergencies, such as postpartum hemorrhage, in order to preserve their public image as knowledgeable and competent. They concluded that poorly skilled nurses were a major hurdle preventing patients and their families from gaining access to proper health care, alongside such barriers as cost of care and distance to health facilities.

The critical role of nursing education became clear in this review of the literature. Formal education remains the ideal conduit through which nurses acquire necessary skills, become socialized to the profession and empowered as a health care force [100]. The global nursing community has united to create and advocate for a universal standard for initial education in order to gain entry into nursing practice [100]. These standards endorse contextually appropriate pedagogy to better prepare nurses for the complex practice reality they will encounter upon entry into the work environment [100]. However, efforts to better prepare nurses to achieve national and local health goals must be matched by well-defined and appropriately legislated nursing practice standards. The case of Botswana highlights the case that producing a qualified workforce is only half the battle. A Family Nurse Practitioner (FNP) program has been in place there since 1973 and intended to prepare nurses who can fulfill a primary care role at an advanced level [101]. A well trained pool of FNPs was envisioned to provide enhanced coverage in a country with no medical school and concomitant severe shortages of physicians. However, both the health system and legislature remain unprepared to absorb this higher level cadre causing instances of confusion about the role of FNPs [101, 102]. With the role of FNPs misunderstood, they are often utilized as nurses or midwives, moving further away from their intended role as primary care providers [102].

Emphasis should be placed on developing a competent, autonomous and dynamic cadre of nursing leaders poised to contribute to organizational decision making. Although management approaches were notably the least examined component of PPMs in our review, their importance cannot be negated. Studies of high performing United States hospitals attributed their success to managers whose commitment to quality of care prompted implementation of processes to attract and retain personnel well-suited to fulfill organizational quality-driven goals and providing “staff the right tools to do their job” [103, 104]. In dynamic clinical milieu, the human resources management practices executed by nurse leaders indirectly influence the quality of health services. However, the capacity of health human resources managers in some low-income regions has been found to be deficient. In 26 sub-Saharan African countries, human resources units within ministries of health were understaffed and subject to frequent turnover [105]. Managers at the ministry of health and district levels were reported not to possess mandated qualifications for their role [105]. Programs like the Global Nursing Leadership Institute and Leadership for Change™, both offered by the International Council of Nurses, present opportunities for nurses in low-income countries to develop leadership skills necessary to overcome complex health systems challenges and drive a nurse-centered agenda. While there is a cost associated with participation in these programs, attendees can apply for sponsorship.

Global health as a discipline has exploded and introduced the need for a new paradigm to define relationships between health personnel in low- and high-income countries. According to the Consortium of Universities for Global Health (CUGH), there are more than 130 universities offering global health programs in Australia, Canada, Denmark, France, Italy, Japan, The Netherlands, Sweden, United States and United Kingdom [106]. Increasingly, schools of nursing are developing dedicated centers for global health scholarship and sending students abroad on international clerkships, but while these enterprises may have been well intentioned, they have not always been without deleterious effects to host institutions and communities in low-income countries [107]. Collaborations between nursing faculty, clinical experts and researchers in low- and high-income countries must be entered into with the view to strengthen health systems in low-income countries; programs should be developed to align with national health goals and adhere to codes of conduct that benefit low-income country partners [108].

One troubling reality emerging from our review is the mistreatment and neglect suffered by some patients while under the care of nurses [26, 27, 29–33]. Unfortunately, disrespect and abuse of patients by some nurses has been a known but largely ignored problem until recently; a symptom of vulnerable health systems unable to respond adequately to multiple pressing needs [109]. However, patients know they want and deserve better. In the case of rural Tanzanian women responding to a discrete choice experiment asking them to rank preferences for place of delivery, respectful treatment by staff was valued higher than other factors, such as distance and cost, in deciding to seek safer facility births [110]. Freedman and Kruk [109] posit that individual actions in violation of patients’ rights occur at the convergence of complex personal, normative and systemic circumstances. Nurses in low-income countries often work in extremely difficult conditions that exert undue pressure on their physical and psychological well-being, which can manifest in poor treatment of patients [109]. A discussion about quality improvement in health services delivery, Freedman and Kruk [109] argue, must include interventions that empower health providers to meet the demand for quality care.

### Limitations

A limitation of this review was the time commitment necessary to analyze the large cache of articles retrieved using the prescribed search strategy. As described previously, we found that enhancing the search with additional terms compromised sensitivity, which meant numerous relevant articles would have been left out. This paradox can be explained by an imprecise alignment between the key words describing our concepts of interest and vocabulary contained in the databases we searched [24]. For example, the term closest in resemblance to the conceptual meaning of *patient care delivery systems* in PubMed was *professional delegation*, which when combined with *nursing* and *low-income countries* did not yield any results. Therefore, we concluded that although it was time consuming, our approach yielded the most pertinent collection of articles for our analysis. It is important to note that we only looked at published articles written in English. As a result, our review could be subject to publication bias. It is possible there are related studies that have not been published or published in a language other than English or indexed in databases not targeted in our search. Nevertheless, our results provide a valuable lens through which capacity building for nurses in low-income countries can be viewed and used to inform future research.

## Conclusion

In low-income countries facing unrelenting health workforce shortages and an overwhelming disease burden, nurses overseeing the bulk of health services delivery require more than an adequate supply of equipment and medications. Functional organizational systems are necessary to support nurses in fulfilling their professional obligations. The discourse on reinforcing the nursing workforce in low-income countries should consider the elements of PPMs, wholly or individually, as a framework around which nursing practice can be structured.

## Abbreviations

ART: Antiretroviral therapy
CUGH: Consortium of universities for global health
FNP: Family nurse practitioner
GAPFON: Global advisory panel on the future of nursing
ICNP: International classification of nursing practice
PPM: Professional practice model
WHO: World Health Organization
